# Comments on "Human Dominant Disease Genes Are Enriched in Paralogs Originating from Whole Genome Duplication"

**DOI:** 10.1371/journal.pcbi.1003758

**Published:** 2014-07-31

**Authors:** Wei-Hua Chen, Xing-Ming Zhao, Vera van Noort, Peer Bork

**Affiliations:** 1 European Molecular Biology Laboratory (EMBL) Heidelberg, Heidelberg, Germany; 2 Department of Computer Science, School of Electronics and Information Engineering, Tongji University, Shanghai, China; 3 Max-Delbrück-Centrum für Molekulare Medizin (MDC), Berlin, Germany; Cornell University, United States of America

We previously showed that monogenic disease genes (MDs) are enriched in duplicates and hypothesized that functional redundancy among duplicates underlies this enrichment [1]. In their comment, Singh et al. refine this enrichment to genes resulting from whole genome duplications (WGDs) [2]; they, furthermore, “could not find any significant enrichment in duplicates in support of possible functional compensation for essential genes” [2] by using gene essentiality data from mouse (transferred to human through orthology).

We appreciate the scientific argument, but we would like to point out that confounding factors and data biases can lead to seemingly opposing conclusions. For example, we carefully considered the duplication age of genes, which is a known confounder in such analyses [3], [4], as well as the use of gene subsets that have known biases such as the mouse essentiality data [3], which, in addition, have issues when conclusions are being transferred to human genes.

First, when using the data of Singh et al. [2] and stratifying small-scale duplicates (SSDs) into old and young groups according to the duplication age relative to WGD, we found that MDs are enriched in old SSDs; limiting this analysis to recessive MDs produced similar results (Figure 1A). In contrast, MDs are depleted in young SSDs (Figure 1B), which is consistent with our hypothesis and with our findings that coexpression decreases with increased duplication age. Thus, when the duplication is old, the ability of the functional copy to compensate for the mutation-carrying malfunctioning copy could be easily disrupted because of random fluctuation in gene expression in a subpopulation; consequently, the gene is associated with a disease, but it will not be purged from the whole population. Therefore, functional compensation can promote the spreading of disease genes in duplicates. However, in young duplicates, the fluctuation in gene expression among duplicates may not be that huge; thus, deleterious mutations could be tolerated, and the corresponding genes are unlikely to associate with any diseases.

**Figure 1:**
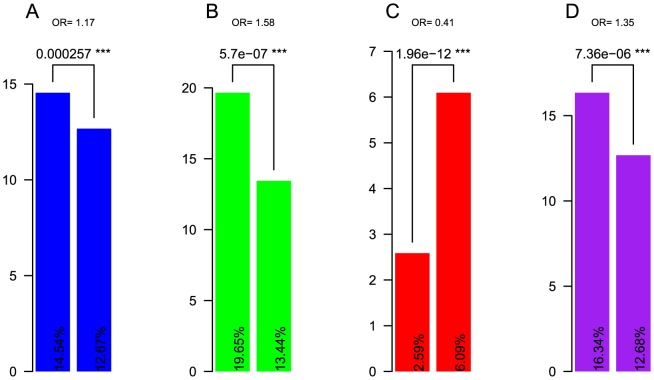
Enrichment of MDs in old SSDs and distinct characteristics of the old SSDs as compared with the young ones. Statistics using data from Singh et al. *P*-values and ORs (odd ratios) are calculated using Fisher's Exact Test (see Dataset S1 for the R code). A. MDs genes are enriched in old duplicates; left: percentage of old SSDs in MDs, right: percentage of old SSDs in all genes. B. Recessive MDs genes are enriched in old SSDs; left: percentage of old SSDs in recessive MDs, right: percentage of old SSDs in all MDs. C. Essential genes are depleted in young SSDs; left: percentage of young SSDs that are essential, right: percentage of young SSDs in tested genes. D. Essential genes are enriched in old SSDs; left: percentage of old SSDs that are essential, right: percentage of old SSDs in tested genes.

Second, mouse essentiality data are biased [5], e.g., towards developmental genes; i.e., they do not correspond to the full spectrum of MDs. Dividing the tested mouse genes into subgroups, the proportion of essential genes in young SSDs is significantly lower than that of singletons (Figure 1C), consistent with functional redundancy among duplicates; however, the opposite is found in old SSDs (Figure 1D). The latter has led to the somewhat counterintuitive conclusion that “duplicates are as essential as singletons” [6], which has been argued against by several follow-up studies [3]–[5]. These results, again, highlight the importance of taking duplication age into consideration. As previous studies suggested, it is not trivial to correct the biases [3]–[5], and hence, conclusions from this data regarding duplications have to be taken with caution. Furthermore, the essentiality status of mouse genes cannot be reliably transferred to human and vice versa. For example, using data from OGEE [7], an online gene essentiality database, 2,322 mouse essential genes have one-to-one orthologs in human; only 476 out of the 2,322 human genes (approximately 20%) were essential according to a genome-wide small interfering RNA (siRNA) experiment [8].

Finally, only less than 30% of the MDs we collected [1] were used in the analyses by Singh et al.; the intersection with the essentiality dataset is even smaller (approximately 18.6% of the MDs used in [1]) because, so far, only less than one-third (approximately 6,400) of mouse genes has been tested for essentiality [9]. Thus, extrapolating any observations on these data to the whole genome would be difficult; for example, some functional signals might only become statistically significant in larger datasets.

Elucidating the molecular basis of human genetic disorders is one of the most important tasks in medical biology. With the relevant data, such as those from genome-wide association studies (GWAS), accumulated at an astonishing speed, integrative and comparative analyses through bioinformatics are much needed. In this regard, Singh et al. did provide an important contribution by refining the enrichment of dominant MDs in duplicates to those derived from WGD. However, we don't believe that they nullified our functional compensation hypothesis with the analyses performed, but they certainly encouraged further studies on more complete datasets, hopefully to be available in the near future.

## Supporting Information

Dataset S1 Raw data and an R script used in this study are available in Dataset S1 as an archive file; readers can use these materials to reproduce our results, including the statistical tests and the figure.
(ZIP)
